# Epstein–Barr virus infection is associated with clinical characteristics and poor prognosis of multiple myeloma

**DOI:** 10.1042/BSR20190284

**Published:** 2019-10-15

**Authors:** Bing Xia, Xi Wang, Ruifang Yang, Li Mengzhen, Kunpeng Yang, Li Ren, Suxia Li, Shuye Wang, Yizhuo Zhang

**Affiliations:** 1 Department of Hematology, Tianjin Medical University Cancer Institute and Hospital, National Clinical Research Center for Cancer, Key Laboratory of Cancer Prevention and Therapy, Tianjin’s Clinical Research Center for Cancer, Tianjin 300060, China; 2 Department of Pediatric oncology, Sun Yat-sen University Cancer Center, State Key Laboratory of Oncology in South China, Collaborative Innovation Center for Cancer Medicine, Guangzhou 510060, China; 3 Department of Clinical laboratory, Tianjin Medical University Cancer Institute and Hospital, National Clinical Research Center for Cancer, Key Laboratory of Cancer Prevention and Therapy, Tianjin’s Clinical Research Center for Cancer, Tianjin 300060, China; 4 Department of Hematology, The First Affiliated Hospital of Harbin Medical University, Harbin 150081, China; 5 Department of Geriatric Hematology, The Second Medical Center of Chinese PLA General Hospital, National Clinical Research Center for Geriatric Diseases, Beijing 100853, China

**Keywords:** correlation analysis, Epstein-Barr virus, multiple myeloma, prognosis

## Abstract

The aim of the present study was to evaluate the relationship of Epstein–Barr virus (EBV) infection and multiple myeloma (MM) and its impact on clinical characteristics and prognosis. Fresh peripheral blood mononuclear cells (PBMCs) from 139 MM patients who had been diagnosed and treated from January 2010 to May 2018 and 50 PBMC samples from healthy donors were obtained. PCR was carried out for detection of EBV-DNA. The results indicated a significantly higher EBV-DNA concentration among 139 MM patients compared with healthy controls (*P*<0.05). Correlation analysis showed that the expression of EBV-DNA was positively correlated with the serum free light chain ratio (sFLCR) and progressive disease (PD)/relapse (*P*<0.05). Especially, in EBV-DNA high-expression MM patients, EBV-DNA concentration for patients with sFLCR ≥100 was higher than that of patients with sFLCR <100. EBV-DNA concentration was higher in patients with disease PD/relapse than those without disease PD/relapse. In univariate analysis, the progress free survival (PFS) was inferior in MM patients with high expression of EBV-DNA, high lactate dehydrogenase (LDH), and high-risk according to mSMART and International Myeloma Working Group (IMWG), stage III according to R-ISS staging, extramedullary lesions, and genetic changes (*P*<0.05). However, in multivariate analysis, LDH, poor karyotype, R-ISS staging, and mSMART were independent prognostic factors for PFS. Taken together, our studies suggest that an association exists between EBV infection and clinical characteristics of MM patients, and EBV infection appears to have a slight impact on the prognosis of MM. However, the results require further validation in other independent prospective MM cohorts.

## Introduction

Multiple myeloma (MM) is the second most common hematological malignancy characterized by monoclonal proliferation of plasma cells that are derived from mature, terminally differentiated B cells [1]. MM patients frequently have depressed levels of polyclonal immunoglobulins and exhibit severe-to-moderate humoral immunodeficiency [2]. Moreover, immune suppressive drug-use during the treatment is another important factor of depressed immunity and subsequent-induced infection [3]. Especially, identification of various infectious factors associated with the progression of MM is crucial in determining disease prognosis and selecting individualized treatment options.

Epstein–Barr virus (EBV) is a member of the *Herpesviridae* family, and EBV infection occurs worldwide. By adulthood, more than 90% individuals are infected by EBV [4]. Individuals in the Chinese population infected with EBV are younger than those in the Western countries [5]. Thus, it is more important to study the relationship between EBV infection and tumor in China. Previous studies showed that EBV interferes with cellular DNA repair mechanisms and could lead to genetic changes in the infected cells [6,7]. EBV, as well as the other herpes viruses, have been associated with different types of B-cell-derived lymphoid malignancies, such as Burkitt’s lymphoma, Hodgkin’s lymphoma, and diffuse large B-cell lymphoma [8]. The B-cell lymphoid malignancies can initiate from a clone of EBV-infected B cells; furthermore, there is evidence that persistent EBV infection may induce disease progression [9]. The correlation between EBV infection and MM is still controversial [10]. Further studies are required to verify the relationship between EBV infection and MM.

Choosing appropriate clinical specimens and laboratory test method is very important for the diagnosis of different EBV infection-related diseases. Real-time PCR (RT-PCR) has the advantages of fast operation and low risk of laboratory pollution [11]. EBV-DNA loads are the most common specimens and have been widely applied in EBV-related disease diagnosis, treatment effect, and prognostic evaluation [12].

In the present study, peripheral blood mononuclear cells (PBMCs) from 139 MM patients were detected by real-time quantitative PCR and 50 healthy donors were selected as control. We evaluated the potential relationship of EBV infection and MM, and its impact on clinical characteristics and prognosis.

## Materials and methods

### Patients

We obtained fresh peripheral blood and isolated mononuclear cells from 139 MM patients who had been diagnosed and treated from January 2010 to May 2018. In addition, our study included 50 fresh peripheral blood samples of age and sex-matched healthy donors that represented the control samples. All patients were staged before treatment using both DS staging system and R-ISS staging system. MM patients were not routinely screened for EBV-DNA at diagnosis in China.

### DNA extraction and PCR

Mononuclear cells from fresh peripheral blood were extracted by lymphocyte isolation fluid (Solarbio, China). EBV nucleic acid amplification fluorescence detection kit was purchased from Da An Gene Co., Ltd. of Sun Yat-Sen University, and it contained the critical positive quality control product, positive product, negative quality control product, and a PCR reaction tube. PCR products were amplified using specific primers (upstream primer, 5′-GTAGAAGGCCATTTTTCCAC-3′; downstream primer, 5′-TTTCTACGTGACTCCTAGCC-3′) and a double fluorescent-labeled probe (5′-(FAM)ACCACCGTGGCCCAGATGG(TAMRA)-3′). The PCR cycling parameters were set as follows: 93°C for 2 min with 1 cycle, 93°C for 45 s and 55°C for 60 s with 60 cycles, followed by 30 cycles of PCR reaction at 93°C for 30 s, and 55°C for 45 s. The reactions were performed in the Bio-Rad CM9600 Real-Time PCR Detection System (Bio-Rad, Hercules, CA). The detection methods, results analysis and quality control methods followed the company’s reagent instructions. EBV-DNA was divided into high expression (>5 × 10^3^ copies/ml) and low expression (<5 × 10^3^ copies/ml) according to the copy number. All PCR reactions were repeated thrice.

### Treatment and follow-up

The diagnosis and therapeutic criteria of MM were identified in accordance with the NCCN guidelines [13]. Follow-up began in January 2010. During induction and consolidation therapy, each course of treatment was followed-up. During the maintenance therapy, the follow-up with the patients was every 3 months. progress free survival (PFS) was measured from the date of diagnosis to disease progression, disease relapse, or to the date of the final follow-up.

### Statistical analysis

The results of EBV-DNA expression level are presented as the mean ± S.D. An unpaired *t* test was used to find the EBV-DNA expression level. Correlation analysis between EBV-DNA expression level and clinical characteristics were analyzed by Spearman’s test. PFS rate was calculated by the Kaplan–Meier method and multivariate survival analysis was performed using the Cox regression model. *P*<0.05 was considered statistically significant. All statistical analyses were evaluated using SPSS24.0 (IBM Corporation, Armonk, NY, U.S.A.).

## Results

### Clinical characteristics

A total of 139 cases were identified. Patients had a median age of 60 years (range: 41–82 years). The group of patients included in the study consisted of 69 men and 70 women, median follow-up was 76 (0–100) months, median PFS was 70 months, all patients were alive and the 5-year PFS was 62.6%. In the MM group, there were 139 patients, including 59 (42.4%) patients with IgG type, 31 (22.3%) patients with IgA type, 28 (20.1%) patients with light chain type, 11 (7.9%) patients with non-secretory type, and 10 (7.3%) patients with IgD type. Fifty-eight cases were treated with bortezomib (41.7%), 23 cases were treated with thalidomine (16.5%), 47 cases were treated with both bortezomib and thalidomine (33.8%), 11 cases were treated without bortezomib and thalidomine (8.0%). Fifteen cases accepted autologous stem cell transplantation (ASCT) as consolidation therapy. Twenty cases were EBV-DNA high-expression patients, 119 cases were EBV-DNA low-expression patients. The clinical features of the EBV-DNA high-expression and EBV-DNA low-expression patients are summarized in Tables 1 and 3. Among 20 cases with EBV-DNA high-expression patients, the median age was 61 years (range: 46–82 years), median PFS was 24 months (3–70), the 5-year PFS was 32.8%, including eight (40.0%) patients with IgG type, two (10.0%) patients with IgA type, four (20.0%) patients with light chain type, two (10.0%) patients with non-secretory type, and four (20.0%) patients with IgD type; ten (50.0%) patients with serum free light chain ratio (sFLCR) ≥100; five (25.0%) patients with extramedullary lesions (adrenal gland, skin, liver, pancreas, and pleura, respectively); among 12 patients (60.0%) with genetic change, four patients with del(17p), one patient with del(17p), lq21amplification and del(13q4), one patient with t(4; 14), one patient with t(14;16), one patient with t(11;14), two patients with del(13q4), two patients with lq21amplification. One patient accepted ASCT as consolidation therapy.

**Table 1:** Clinical features of patients with high expression level of EBV-DNA patients

P	Age (year)/sex	Classification	DS/R-ISS stage	Msmart/ IMWG	sFLCR	EBV-DNA (copies/ml)	Extramedullary lesions	Genetics change	Theraputic	Therapeutic evaluation	PFS (months)
1	82/F	IgG-k	III/II	MR/LR	<100	6.0 × 10^3^	No	No	T	PR	60
2	69/M	IgG-k	II/I	MR/LR	<100	8.4 × 10^3^	Yes	No	T	PR	30
3	60/F	IgG-λ	I/II	MR/MR	<100	1.0 × 10^4^	Yes	Yes	B	SD	16
4	69/M	IgD-λ	III/II	MR/LR	≥100	1.4 × 10^4^	No	No	B	PD	7
5	46/M	k	II/II	LR/LR	<100	5.0 × 10^3^	No	No	B + T	CR	12
6	59/M	—	II/I	MR/MR	<100	8.1 × 10^3^	No	Yes	B	VGPR	6
7	61/F	λ	III/III	MR/LR	<100	1.1 × 10^4^	No	No	B	PD	22
8	67/F	IgG-λ	III/II	MR/MR	≥100	6.8 × 10^3^	Yes	Yes	T	PD	12
9	56/F	IgG-k	III/II	MR/MR	≥100	1.2 × 10^4^	No	Yes	B	PD	3
10	68/F	IgD-λ	III/III	HR/HR	≥100	1.3 × 10^4^	No	Yes	B	Replase	10
11	68/F	IgD-λ	III/III	HR/HR	≥100	1.8 × 10^4^	No	Yes	B	Replase	24
12	53/F	IgA-λ	III/III	HR/HR	<100	9.7 × 10^3^	No	Yes	B	PD	12
13	67/F	IgA-k	III/III	HR/MR	≥100	1.2 × 10^4^	No	Yes	Without B + T	Replase	36
14	61/M	IgG-k	II/II	MR/MR	≥100	8.3 × 10^3^	No	Yes	T + ASCT	Replase	70
15	58/M	IgG-λ	III/III	HR/HR	<100	7.2 × 10^3^	No	Yes	T	PR	12
16	72/M	k	III/II	MR/LR	≥100	5.1 × 10^3^	No	Yes	B + T	SD	18
17	67/M	IgG-λ	III/III	HR/HR	≥100	9.0 × 10^3^	No	Yes	B	PD	6
18	61/M	λ	III/II	LR/MR	<100	6.1 × 10^3^	No	No	B + T	CR	18
19	61/M	—	I/I	MR/LR	<100	5.0 × 10^3^	Yes	No	T	PR	15
20	53/M	IgD-λ	III/II	LR/LR	<100	1.2 × 10^4^	Yes	No	B	VGPR	30

Abbreviations: CR: complete remission; HR: high risk; IMWG: International Myeloma Working Group; LR: low risk; MR: minimal response; PD: progressive disease; PR: Partial response; VGPR: very good partial response.

### DNA levels of EBV in MM patients and healthy individuals in control group

**Figure 1:**
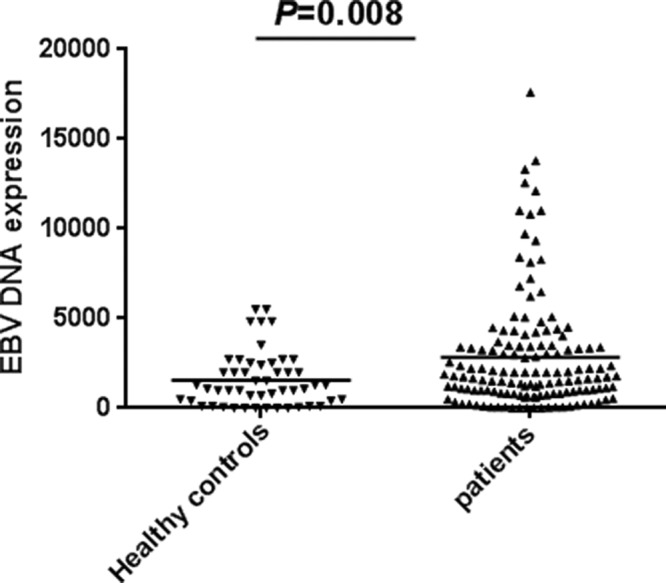
The expression level of EBV-DNA between healthy controls and MM patients

The results of real-time PCR showed that the expression level of EBV-DNA in MM patients is higher than the expression level of EBV-DNA in the healthy control group (2823 ± 272.8 copies vs 1561 ± 214.9 copies, *P*=0.008) (Figure 1).

### Correlation analysis between EBV-DNA expression level and clinical characteristics in MM patients

**Figure 2:**
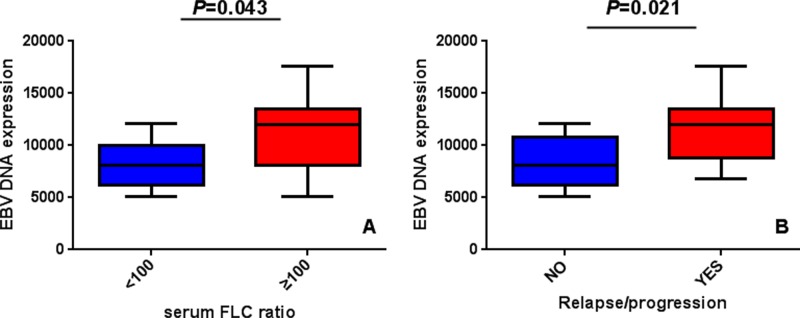
The expression level of EBV-DNA between different subgroups of MM patients (**A**) Among high EBV-DNA expression level group, sFLCR ≥100 patients have higher EBV-DNA expression level compared with sFLCR <100 patients; (**B**) PD/relapse patients have higher EBV-DNA expression level compared with non-PD/relapse patients.

Correlation analysis showed that sFLCR (*rs*=0.237; *P*=0.029) and progressive disease (PD)/relapse (*rs*=0.285; *P*=0.008) positively correlated with EBV-DNA expression (Table 2). Other clinical characteristics including age, gender, staging, risk stratification, genetic change, extramedullary lesions, therapeutic, the level of lactate dehydrogenase (LDH), albumin, β2-MG, HGB, and ASCT were not correlated with EBV-DNA expression. Meanwhile, among the high-expression EBV-DNA group, sFLCR ≥100 and PD/relapse patients had higher EBV-DNA expression level compared with sFLCR <100 (*P*=0.043) and non-PD/relapse (*P*=0.021) patients (Figure 2).

**Table 2:** Correlation analysis between EBV-DNA expression level and clinical characteristics in MM patients

Factors	EBV-DNA expression		rs	*P-value*
	Low (%)	High (%)		
**Gender**			−0.017	0.842
Male	60 (50.4%)	9 (45.0%)		
Female	59 (49.6%)	11 (55.0%)		
**Age (years)**			0.079	0.353
<60	58 (48.7%)	8 (40.0%)		
≥60	61 (51.3%)	12 (60.0%)		
**HGB (g/l)**			0.029	0.731
<115	75 (63.0%)	13 (65.0%)		
≥115	44 (37.0%)	7 (35.0%)		
**sFLCR**			0.237	0.029
<100	104 (87.4%)	10 (50.0%)		
≥100	15 (12.6%)	10 (50.0%)		
**Serum creatinine (umol/l)**			0.064	0.454
<177	98 (82.4%)	16 (80.0%)		
≥177	21 (17.6%)	4 (20.0%)		
**LDH (U/l)**			−0.010	0.910
<248	101 (84.9%)	18 (90.0%)		
≥248	18 (15.1%)	2 (10.0%)		
**β2-MG (mg/l)**			−0.023	0.786
<5.5	75 (63.0%)	14 (70.0%)		
≥5.5	44 (37.0%)	6 (30.0%)		
**Albumin (g/l)**			0.064	0.454
<35	30 (25.2%)	7 (35.0%)		
≥35	89 (74.8%)	13 (65.0%)		
**Genetics change**			−0.019	0.827
No	42 (35.3%)	8 (40.0%)		
Yes	77 (64.7%)	12 (60.0%)		
**Extramedullary lesions**			−0.013	0.876
No	89 (74.8%)	15 (75.0%)		
Yes	30 (25.2%)	5 (25.0%)		
**DS stage**			0.037	0.665
stage I	19 (16.0%)	2 (10.0%)		
stage II	16 (13.4%)	4 (20.0%)		
stage III	84 (70.6%)	14 (70.0%)		
**R-ISS stage**			0.007	0.936
stage I	30 (25.2%)	3 (15.0%)		
stage II	55 (46.2%)	13 (65.0%)		
stage III	34 (28.6%)	4 (20.0%)		
**mSMART**			0.019	0.823
Low	30 (41.1)	5 (41.7%)		
Middle	24 (32.9%)	4 (33.3%)		
High	22 (26.0)	3 (25.0%)		
**IMWG**			0.089	0.299
Low	21 (17.6%)	3 (15.0%)		
Middle	72 (60.5%)	11 (55.0%)		
High	26 (21.9%)	6 (30.0%)		
Theraputic*			−0.071	0.405
B	50 (42.0%)	8 (40.0%)		
T	17 (14.3%)	6 (30.0%)		
B + T	42 (35.3%)	5 (25.0%)		
without B and T	10 (8.4%)	1 (5.0%)		
**ASCT**			0.112	0.188
No	108 (88.3%)	19 (95.0%)		
Yes	14 (11.7%)	1 (5.0%)		
**Therapeutic evaluation**			0.076	0.376
CR/nCR	35 (29.7%)	7 (35.0%)		
VGPR	8 (6.8%)	2 (10.0%)		
PR/SD/PD	75 (63.6%)	11 (55.0%)		
**Follow-up**			0.285	0.008
Alive	99 (83.2%)	10 (50.0%)		
Alive with progression	15 (12.6%)	6 (30.0%)		
Alive with relapse	5 (4.2%)	4 (20.0%))		

**Note:** *B was bortezomib group; T was thalidomine group; B+T was bortezomib and thalidomine.Abbreviation: IMWG: International Myeloma Working Group.

### Survival analysis

**Figure 3:**
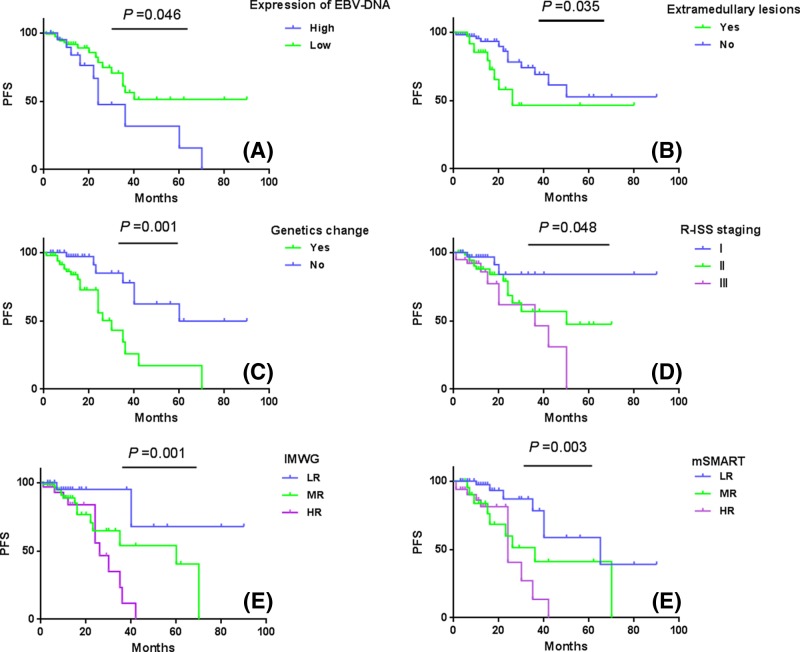
Univariate analysis for survival of MM patients (**A**) The expression level of EBV-DNA for PFS in MM patients; (**B**) extramedullary lesions for PFS in MM patients; (**C**) genetic change for PFS in MM patients; (**D**) R-ISS staging for PFS in MM patients; (**E**) IMWG for PFS in MM patients; (**F**) mSMART for PFS in MM patients.

In univariate analysis (Figure 3), compared with the low-expression EBV-DNA group, the high-expression EBV-DNA group had lower PFS (*P*=0.046). In addition, the PFS rates were inferior in MM patients with high LDH (*P*=0.014), high-risk by mSMART (*P*=0.003), and International Myeloma Working Group (IMWG) (*P*=0.001), stage III by R-ISS staging (*P*=0.048), extramedullary lesions (*P*=0.035), and genetic change (*P*=0.001). In multivariate analysis, LDH (hazard ratio [HR]: 0.404; 95%CI: 0.146–1.116; *P*=0.048), genetic change (HR: 0.295; 95% CI: 0.105–0.825; *P*=0.020), R-ISS staging (HR: 0.462; 95% CI: 0.192–1.111; *P*=0.045), and mSMART (HR: 2.478; 95% CI: 1.006–6.103; *P*=0.049) were independent predictors for PFS (Table 3).

**Table 3:** Cox multivariate regression analysis for PFS in MM patients

Factors	PFS	
	HRs (95% CI)	*P-value*
The expression level of EBV-DNA	0.583 (0.241–1.407)	0.230
LDH	0.404 (0.146–1.116)	0.048
R-ISS staging	0.462 (0.192–1.111)	0.045
Extramedullary lesions	0.409 (0.097–1.722)	0.223
Genetics change	0.295 (0.105–0.825)	0.020
mSMART	2.478 (1.006–6.103)	0.049
IMWG	1.080 (0.417–2.798)	0.874

## Discussion

Several case reports have consistently demonstrated an association between EBV and MM. As early as 1986, there was a case report of EBV positivity after kidney transplantation in patients with secondary MM [14]. In 1995, a case was reported of an AIDS patient who developed MM with a particularly aggressive course that suggested that in the presence of EBV RNA in the plasma cells, EBV plays an etiological role in the development of MM [15]. Another case was described of a patient who developed MM after renal transplantation. EBV-RNA was demonstrated in the neoplastic cells implicating this virus genome in the pathogenesis of the post-transplantation lymphoproliferative disorder [16]. However, most reported patients with EBV-positive plasmacytoma are in a state of immunosuppression. Whether EBV infection promoted the occurrence and progress of MM is controversial. The finding that EBV-DNA concentrations of PBMC in patients with MM were significantly higher than that in individuals in the general population suggests that an association exists. However, our data indicated that the positive rate of EBV-DNA was not statistically significant between patients and the normal controls. Immunodeficient patients, such as those with an EBV infection and those undergoing immunosuppressive drug therapy for transplantation immunosuppressive drug therapies or transplantations, have a slightly increased risk of developing EBV-associated plasmacytoma, and immune regulatory events triggered by the monoclonal myeloma Ig establish and maintain the immunodeficiency in MM patients [17]. The latent infection rate of EBV is generally higher in healthy people [18].

Our research also indicated that the EBV-DNA concentration of PBMC in MM patients was significantly correlated with disease progression/relapse (*P*<0.05). Likewise, in MM patients with EBV high expression, EBV-DNA concentration was higher in patients with progression/relapse than those without progression/relapse. Similarly, Yan et al. [19] investigated the correlation between EBV infection and solitary plasmacytoma (SP) in 46 patients. The results indicated that EBER-positive patients were more likely to show disease progression (relapse/progression to MM) than EBER-negative patients. Furthermore, we found that EBV-DNA was clearly related to sFLCR, and in the EBV high-expression group, EBV-DNA concentration in cases where sFLCR >100 was higher than that in the sFLCR <100 cases. The sFLCR at initial diagnosis has been widely accepted and applied as a new parameter for the prognosis of patients with myeloma [20]. Paiva et al. [21] found that patients who obtained stringent complete response (sCR) with normal sFLCR had longer PFS than those with abnormal sFLCR, suggesting that sFLCR is an independent prognostic factor of MM. But the related mechanisms remain to be further investigated.

Follow-up data is available for 139 MM patients, all of whom were alive during the last follow-up. The results showed that compared with the low-expression EBV-DNA group, the high-expression EBV-DNA group has lower PFS (*P*=0.047), but multivariate analysis indicated that EBV-DNA expression was not the independent prognostic factor. A previous study has suggested that EBV positivity is of importance when the differential diagnosis exists between MM and plasmablastic lymphoma (PBL), which is an aggressive malignant B-cell neoplasm. PBL patients (60–75%) have EBV infections and usually die within 1 year [22]. However, the clinical course of MM is indolent. To our knowledge, this is the first study that suggests the role of EBV infection in affecting prognosis of MM. More definitive results need to be confirmed by large sample prospective studies. Similar to previous studies, the present study also showed LDH, genetic change, and R-ISS staging. mSMART and IMWG were independent predictors of PFS [23].

## Conclusion

In conclusion, the current study suggests that an association may indeed exist between EBV infection and MM. In addition, our results also indicate that EBV infection is closely related to the clinical characteristics and prognosis of MM patients.

## Informed Consent

Informed consent was obtained from all individual participants included in the study.

## Abbreviations

ASCT: autologous stem cell transplantation
DNA: deoxyribonucleic acid
EBV: Epstein–Barr virus
FLC: free light chain
HR: hazard ratio
IMWG: International Myeloma Working Group
LDH: lactate dehydrogenase
MM: multiple myeloma
NCCN: The National Comprehensive Cancer Network
PBL: plasmablastic lymphoma
PBMCs: peripheral blood mononuclear cell
PD: progressive disease
PFS: progress free survival
RNA: ribonucleic acid
RT-PCR: real-time PCR
sCR: stringent complete response
sFLCR: serum free light chain ratio
